# P-1175. Discrepancies Between Bacteriophage Plaque Assays and Time-Kill Analyses in Daptomycin Non-Susceptible Methicillin-Resistant Staphylococcus aureus

**DOI:** 10.1093/ofid/ofaf695.1368

**Published:** 2026-01-11

**Authors:** Sean R Van Helden, Callan Bleick, Michael J Rybak

**Affiliations:** Wayne State University, Eugene Applebaum College of Pharmacy and Health Sciences, Detroit, Michigan; Anti-Infective Research Laboratory, College of Pharmacy and Health Sciences, Wayne State University, Detroit, MI, Wayne State University School of Medicine, Department of Microbiology and Immunology, Detroit, MI, Detroit, Michigan; Eugene Applebaum College of Pharmacy and Health Sciences, Detroit, Michigan

## Abstract

**Background:**

Bacteriophage (phage) therapy is a promising adjunct to antibiotics in treating methicillin-resistant *Staphylococcus aureus* (MRSA), including daptomycin non-susceptible (DNS) strains. Phage activity against specific bacterial strains is most frequently determined using the double agar overlay plaque-forming assay (plaque assay). Although this assay is often utilized in laboratory settings for determining phage activity, the conditions under which it is performed may not correspond to those of other in vitro assays or models and thus may compromise its ability to reliably predict phage efficacy in these applications. In this study, we describe discrepancies observed between plaque assays and time-kill analyses (TKAs) across multiple phages and DNS MRSA strains.

**Figure:**
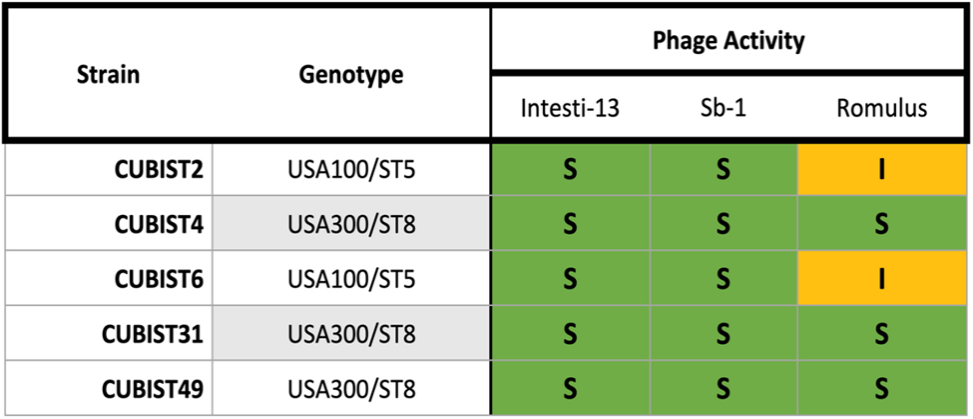

Activity of bacteriophages Intesti-13 (Kayvirus), Sb-1 (Kayvirus), and Romulus (Silviavirus) as determined by the double agar overlay plaque-forming assay against various daptomycin non-susceptible methicillin-resistant Staphylococcus aureus strains. Activity was classified as susceptible (S, >107 PFU/mL), intermediate (I, 103-107 PFU/mL), or resistant (R, <103 PFU/mL). Each experiment was performed in duplicate. S. aureus genotypes were categorized based on pulsed-field gel electrophoresis and multilocus sequence typing.

**Figure:**
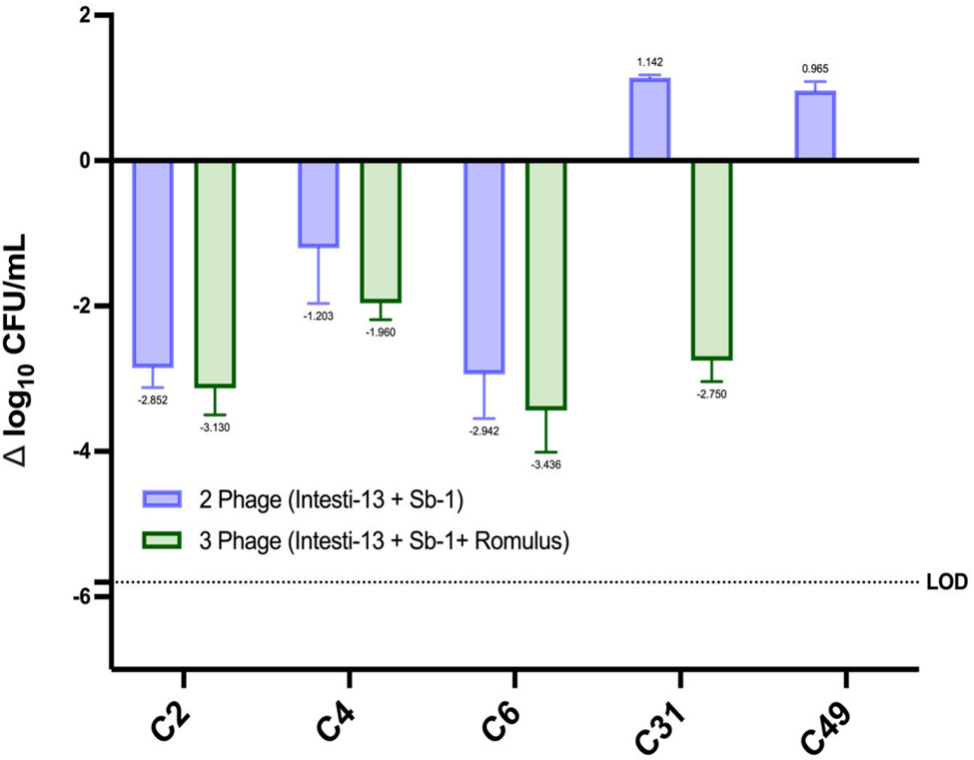

Time-kill analyses of two-phage cocktail of Kayvirus Instesti-13 and Sb-1 (blue) and three-phage cocktail of Intesti-13, Sb-1, and Silviavirus Romulus (green) against daptomycin non-susceptible methicillin-resistant Staphylococcus aureus strains. Change in bacterial burden was measured as the change in log10 CFU/mL. Each experiment was performed in duplicate. Error bars represent standard deviation.

**Methods:**

We compared the activity of two *Kayvirus* phages, Intesti-13 and Sb-1, as well as the *Silviavirus* phage Romulus, against five DNS-MRSA strains from the Cubist repository (C2, C4, C6, C31, and C49) using both plaque assays and TKAs. Plaque assays were conducted via double agar overlay method using high titer (10^9^ PFU/mL) stocks of each phage against lawns of each bacterial strain of interest. Subsequently, TKAs consisting of 2- and 3-phage cocktails were performed at a multiplicity of infection (MOI) of 1.0. Bacterial reduction (Δ log_10_ CFU/mL) was assessed to determine phage activity.

**Results:**

Plaque assays revealed potent activity of both Intesti-13 and Sb-1 against all DNS-MRSA strains. Romulus had intermediate activity (i.e., faint plaquing) against C2 and C6 with increased activity against all other strains tested. In 24h TKAs, the 2-phage cocktail (Intesti-13 + Sb-1) achieved adequate killing against strains C2, C4, and C6 (-2.85, -1.20, and -2.94 log_10_ CFU/mL, respectively). However, against strains C31 and C49, bacterial growth was observed (+1.14 and +0.97 log_10_ CFU/mL, respectively), contradicting the findings of the aforementioned plaque assay. The addition of Romulus to the 2-phage cocktail generally improved efficacy, particularly in C31.

**Conclusion:**

We described discrepancies in observed phage activity in plaque assays vs in vitro planktonic TKAs, suggesting that plaque assays may be a poor predictor of phage activity in other applications.

**Disclosures:**

Michael J. Rybak, PharmD, PhD, MPH, Abbvie: Grant/Research Support|Innoviva: Grant/Research Support|Melina: Grant/Research Support|Merck: Grant/Research Support|Shionogi: Grant/Research Support

